# Anticancer, Enhanced Antibacterial, and Free Radical Scavenging Potential of Fucoidan- (*Fucus vesiculosus* Source) Mediated Silver Nanoparticles

**DOI:** 10.1155/2021/8511576

**Published:** 2021-09-30

**Authors:** S. Rajeshkumar, Eman F. Aboelfetoh, Sri Renukadevi Balusamy, Daoud Ali, Mohammed H. A. Almarzoug, Jule Leta Tesfaye, Ramaswamy Krishnaraj

**Affiliations:** ^1^Department of Pharmacology, Saveetha Dental College and Hospital, SIMATS, Chennai, 600077 Tamil Nadu, India; ^2^Chemistry Department, Faculty of Science, Tanta University, Tanta 31527, Egypt; ^3^Department of Food Science and Biotechnology, Sejong University, Gwangjin-gu, Seoul 05006, Republic of Korea; ^4^Department of Zoology, College of Science, King Saud University, PO Box 2455, Riyadh 11451, Saudi Arabia; ^5^Centre for Excellence-Indigenous Knowledge, Innovative Technology Transfer and Entrepreneurship, Dambi Dollo University, Ethiopia; ^6^Department of Physics, College of Natural and Computational Science, Dambi Dollo University, Ethiopia; ^7^Department of Mechanical Engineering, Dambi Dollo University, Ethiopia

## Abstract

The present research displays the green synthesis of stable silver nanoparticles (AgNPs). The aqueous solution of Fucoidan from *Fucus vesiculosus* source (brown marine algae) is used as a reducing and capping agent. UV-Vis spectroscopy, XRD, FT-IR, SEM, EDX, and TEM with selected area electron diffraction are used to characterize the synthesized silver nanoparticles (AgNPs). The synthesized AgNPs exhibit a surface plasmon resonance at 430 nm after 24 h. The characterization results showed that AgNPs are crystalline in nature and exhibit mostly spherical shapes with an average diameter of 4-45 nm. Silver nanoparticles showed effective antibacterial activity against representative pathogens of bacteria. The activities of commercial antibiotics were enhanced by impregnation with the synthesized AgNPs. It also shows good fungicidal and anticancer activity against liver and lung cell lines and shows significant antioxidant efficacy (84%) at 10 *μ*g/ml AgNP concentration against DPPH. The utilization of environmentally synthesized AgNPs offers numerous benefits of ecofriendliness and compatibility for biomedical applications.

## 1. Introduction

The noble metal nanoparticles have gained great interest in a number of studies due to their potential applications in medical, optical, electronic devices, and water treatment. The most prominent challenge is how to control their sizes and shapes. For this purpose, a large number of reports have been published for the synthesis of metal nanoparticles of diverse structures [1]. The silver nanoparticles have become a comprehensive research point owing to their broad range of applications as disinfectant agents, catalyst, biosensor, and water treatment [2]. Various approaches were made for synthesis of AgNPs such as chemical reduction, electrochemical techniques, photochemical reduction, sonochemical, microwave, and radiation-assisted process [3]. Among these methods, the chemical reduction method is the most frequently used but remains costly and employs risky substances, such as organic solvents and harmful reducing agents, e.g., sodium borohydride, hydrazine, and N,N-dimethyl formamide. Furthermore, surface passivation and capping agents are generally added to the reaction system to avoid aggregates formation of the nanoparticles [3]. The state-of-the-art studies have focused on the green synthesis approaches to avoid utilization of highly toxic materials. These approaches emphasize on the utilization of ecofriendly, cost-effective, and biocompatible reducing agents for the synthesis of AgNPs, which gives the synthesized AgNPs sufficient stability in the strong electrolytic and pH conditions for therapeutic application [4]. Various biological organisms have emerged as simple and viable substitutes to obtain AgNPs such as bacteria, yeast, fungi, algae, and plants [4–7]. Polysaccharides acquired from marine algae include fucoidan, alginate, ascophyllan, agar, and carrageenan as phycocolloids have been used for decades in medicine and pharmacy [8–15]. Fucoidan (sulfated fucan) is one of nonstarch polysaccharide which is soluble in water. It has complicated chemical structure and found in brown sugars such as galactose, xylose, and mannose. Fucoidan is derived from marine brown algae, containing large quantities of L-fucose and sulfated including *Fucus vesiculosus* [15–20]. Due to the wide spectrum of activity of fucoidan in biological systems including antitumor, immunomodulatory, antibacterial, antiviral, anti-inflammatory, anticoagulant, and antithrombotic effects [20–25], the aim of the present study is to synthesize AgNPs using the commercially available fucoidan source of *Fucus vesiculosus* (Fv-fucoidan) as a reducing and capping agent and to explore the potential antimicrobial, antioxidant, and anticancer activities of the synthesized AgNPs.

## 2. Materials and Methods

Fucoidan from *Fucus vesiculosus* source (brown marine algae) and silver nitrate was purchased from Sigma Aldrich, India, and the standard antibiotics and media were purchased from HiMedia laboratories, Mumbai, India. The bacterial strains used in the present study were obtained from Micro Labs, Tamil Nadu, India (*Bacillus* sp., *Serratia pnematodiphila*, *Streptococcus* sp., and *Klebsiella pneumoniae*), and Micro Labs, Chandigarh, India (*Bacillus subtilis* and *Klebsiella planticola*).

### 2.1. Synthesis and Characterization of AgNPs

Colloidal AgNPs were synthesized by reducing the silver nitrate in an aqueous solution of fucoidan from *Fucus vesiculosus* source (brown marine algae). Briefly, fucoidan-stabilized AgNPs were synthesized by mixing aqueous solutions of fucoidan (10 ml) and silver nitrate (100 ml, 1 mM) in an Erlenmeyer flask at room temperature. The flask was kept in the orbital shaker at 300 rpm to homogenize the resulting solution for 20 min. The reduction of Ag+ to AgNPs was regularly checked by the UV-visible spectra. The analysis was done using UV-visible spectrophotometer (Perkin Elmer) in the wavelength range 350–650 nm.

The synthesized AgNPs were purified using deionized water with continuous centrifugation, collected, and dried in hot air oven at 80°C for 2 hours. The dried AgNPs were characterized by various techniques. The crystalline nature was studied by XRD (Bruker, Karlsruhe, Germany), and the functional groups responsible for reduction of silver ions and stabilization of the formed AgNPs were studied by Fourier transform infrared (FT-IR) spectroscopy (Perkin Elmer). To examine the size and shape of AgNPs, the transmission electron microscope (TEM) (Hitachi S-4500) and scanning electron microscope (SEM) (Philip model CM 200) were used. The elemental analysis was determined by energy-dispersive X-ray spectroscopy (EDS) attached to the SEM [26–28].

### 2.2. Antibacterial Assessment

The antibacterial activity of fucoidan-stabilized AgNPs was examined against six bacterial strains (*Bacillus* sp., *Serratia pnematodiphila*, *Streptococcus* sp., *Klebsiella pneumoniae*, *Bacillus subtilis*, and *Klebsiella planticola*). The standard antibiotic disks were purchased from HiMedia laboratories (Mumbai, India). Two different diffusion methods were applied [27–30] to assess the antibacterial activity.

### 2.3. Agar Well Diffusion Method

The antibacterial activity of the synthesized AgNPs were explored against Gram-negative (*Serratia pnematodiphila*, *Klebsiella pneumonia*, and *Klebsiella planticola*) and Gram-positive (*Streptococcus* sp., *Bacillus subtilis*, and *Bacillus* sp.) bacteria by adopting the agar well diffusion method [10]. Roughly, 20 ml of sterilized and cooled Mueller-Hinton agar medium was filled with sterile Petri dishes and permitted to solidify at room temperature. The overnight growth test organisms were spread over the agar medium by a sterile cotton swab for each test, and then, the wells were made using a sterile polystyrene tip. Diverse concentrations of AgNPs (25, 50, and 75 *μ*l) were added to the wells. The AgNP-inoculated plates were incubated for 24 h at 37°C. After that, the inhibition zone around the well was calculated and recorded. The tests were done in triplicates [31–34].

### 2.4. Disk Diffusion Method

Disk diffusion method was used to evaluate the in vitro enhanced antibacterial activity of diverse antibiotics (ampicillin, tetracycline, novobiocin, penicillin, kanamycin, gentamicin, chloramphenicol, streptomycin, and ciprofloxacin) against the clinical isolates of bacteria (such as *B. subtilis*, *Bacillus* sp., *S. nematodiphila*, *K. planticola*, *K. pneumoniae*, and *Streptococcus* sp.). To determine the mutual effect, each standard antibiotic disks was further impregnated with 25 *μ*l of the freshly prepared AgNPs. The Petri dishes containing 20 ml Mueller-Hinton agar (MHA) were swabbed with 24 h culture of bacterial strains. Standard sterile antibiotic disks are known as positive control, and antibiotics disks impregnated with AgNPs were placed onto the MHA medium inoculated with pathogenic bacterial isolates. The inoculated plates were then incubated at room temperature for 24 h. After the incubation, the zone of inhibition was measured, and the assays were performed in triplicates. The enhancement in the fold area in the zone of inhibition was evaluated by calculating the mean surface area of the inhibition zone generated by an antibiotic (*a*) and AgNPs impregnated with an antibiotic (*b*). The fold increase area was calculated by the following equation.

(1) $$\begin{array}{c} \frac{\left(b^{2}-a^{2}\right)}{a^{2}}, \end{array}$$

where *a* refers to the inhibition zones for antibiotic alone and *b* refers to the AgNPs impregnated with antibiotic, respectively [11].

### 2.5. Antifungal Susceptibility by the Well Diffusion Method

Several pathogenic impacts of fungi have been stated in plants and animals, as well as humans. The opportunistic infections are caused by fungi such as *Aspergillus niger*, *Aspergillus fumigatus*, *Candida* sp., and *Aspergillus flavus*. Inoculum suspensions were prepared by scratching the surface of the colonies via an antiseptic needle, and the fungal spores were blended with 10 ml sterilized distilled water. Each fungal suspension was swabbed consistently using sterile cotton swabs on sterilized Potato Dextrose Agar (PDA) plates. With the help of a sterilized polystyrene tip, about 3 wells (5 mm diameter) were prepared. Diverse concentrations of AgNPs solution (50 *μ*l, 100 *μ*l, and 150 *μ*l) were added to each well on all plates. Then, the plates were incubated at 37°C for 48-78 h. A clear inhibition zone around the wells was detected. For each organism, the inhibition zone diameter was measured (in millimeter).

### 2.6. Anticancer Activity of AgNPs against HepG2 and A549 Cell Lines

#### 2.6.1. Cell Viability Test

The in vitro cytotoxic effect of the synthesized AgNPs on HepG2 and A549 cell lines were evaluated by MTT assay [11]. Briefly, the cell lines were plated separately in 96 well plates (1 × 104 cells/well) and incubated for 24 h at 37°C in 5% CO_2_. Afterwards, the cells were washed twofold with 100 *μ*l of serum-free medium and starved for 1 h in CO_2_ incubator. Subsequently, the cells were treated with various concentrations of AgNPs in the range of 1-100 *μ*g/ml and again warmed at 37°C in CO_2_ incubator. After 24 h incubation, MTT (0.05 mg/ml) was added in every well and again incubated for 4 h. The MTT-containing medium was thrown away and washed with 200 *μ*l phosphate buffer saline solutions. Then, the crystals were dissolved by adding 100 *μ*l of DMSO and mixed well. The appearance of purple blue formazan dye was measured in a microplate reader (570 nm). The cytotoxicity of AgNPs is analyzed using the GraphPad Prism 5 software.

### 2.7. Antioxidant Activity of AgNPs (DPPH Radical Scavenging Activity)

The formation of free radicals or the deficient removal of reactive oxygen species can develop oxidative damage to biomolecules. These damage leads to numerous sicknesses for human, such as tumor, atherosclerosis, diabetes, maturing, and other degenerative disorders [12]. DPPH (2,2-diphenyl-2-picrylhydrazyl) is a stable free radical that accepts an electron or hydrogen radical from the antioxidant compound and gets reduced to a stable diamagnetic molecule. The reduction of DPPH is associated with color change from pink to yellow. The scavenging ability of DPPH free radicals by fucoidan extract-mediated AgNPs and vitamin C was used as standard as mentioned in the previous study [13]. The percentage of inhibition was calculated by utilizing the following equation:

(2) $$\begin{array}{c} \%\text{Inhibition}=\left(\frac{\text{absorbance of control}-\text{absorbance of test sample}}{\text{absorbance of control}}\right)\times 100. \end{array}$$

## 3. Results and Discussion

### 3.1. Visual Inspection and UV-Visible Spectroscopy of AgNPs

**Figure 1:**
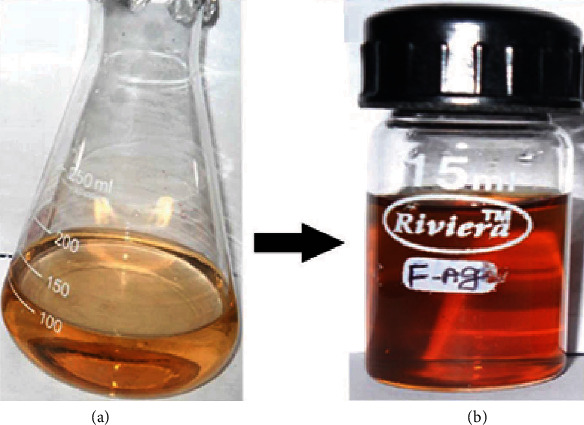
Fucoidan- (*F. vesiculosus*) mediated AgNPs. (a) Initial reaction and (b) after 24 h final color change reaction.

**Figure 2:**
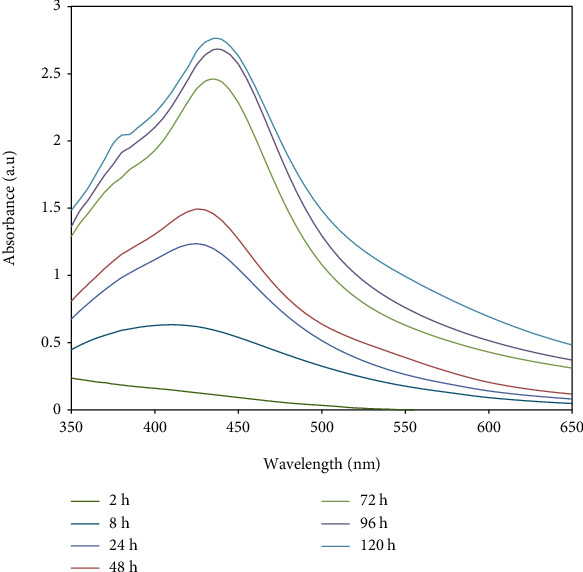
UV-Vis spectra of fucoidan-stabilized AgNPs at different time intervals. *X*-axis indicates the wavelength in nm and *Y*-axis indicates the absorbance of nanoparticles.

The formation of AgNPs by aqueous solution of fucoidan at room temperature was affirmed by visual examination. As appeared in Figure 1, the color of the reaction mixture change from yellow to brown which shows the generation of silver nanoparticles, due to the reduction of Ag ions into AgNPs through the active molecules present in the fucoidan extract. This color is credited due to the excitation of surface plasmon spectra (SPR). Moreover, the formation of AgNPs was followed by measuring the UV-visible absorbance at various time intervals in the range of 350–650 nm (Figure 2).

### 3.2. X-Ray Diffraction Analysis

**Figure 3:**
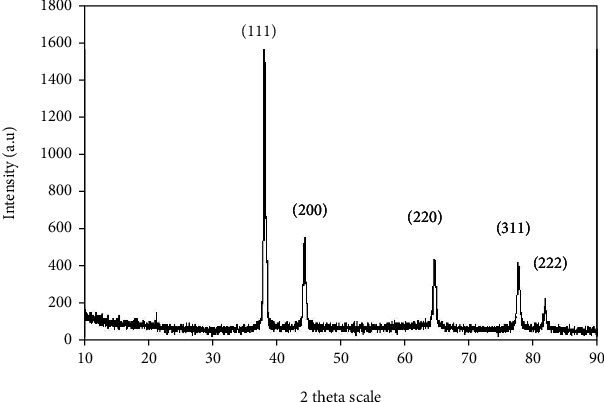
XRD pattern of the synthesized AgNPs. *X*-axis shows the 2-theta scale, and *Y*-axis indicates the intensity of the silver nanoparticles.

The crystalline nature of AgNPs was confirmed by XRD (Figure 3). The diffraction patterns showed four distinct peaks at 2*θ* = 38.15°, 44.30°, 64.53°, and 76.96°. These peaks can be indexed to the (111), (200), (220), and (311) reflection planes which predicts the face centered cubic structure (fcc) of AgNPs in agreement with the previous study.

### 3.3. Transmission Electron Microscopy

**Figure 4:**
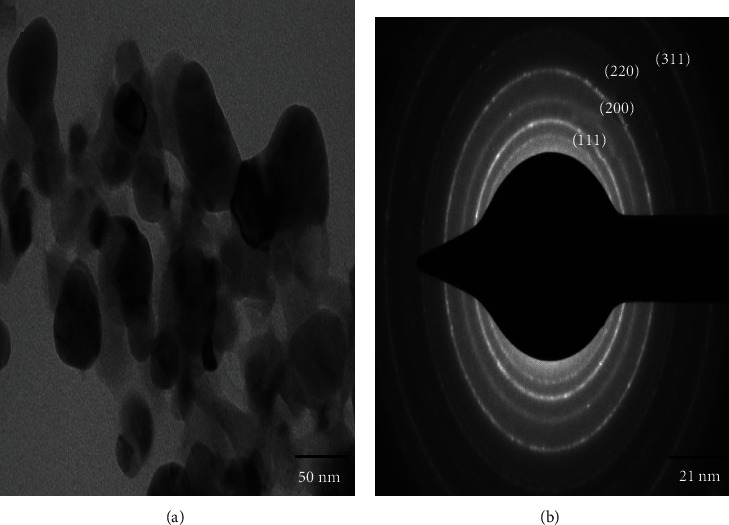
(a) TEM micrograph of AgNPs and (b) SAED pattern.

The TEM image of AgNPs showed monodisperse nanoparticles with spherical shape in the size ranges from 4 to 45 nm (Figure 4(a)). Moreover, the crystalline nature of the nanoparticles is evidenced by the selected area electron diffraction (SAED) patterns with bright circular spots matching to (111), (200), (220), and (311) planes. The SAED pattern results stay in concordant good agreement with the previous study [14].

### 3.4. Energy-Dispersive X-Rays (EDX)

**Figure 5:**
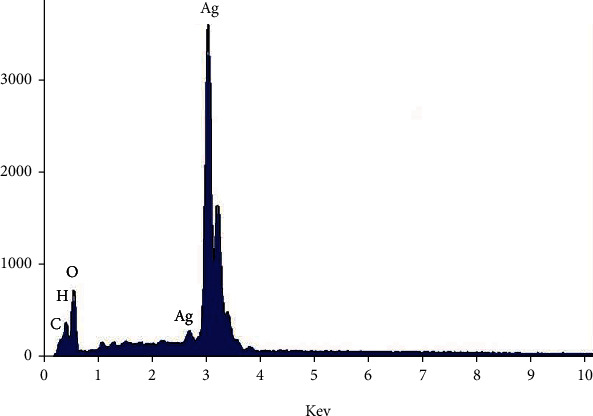
EDX spectrum of the synthesized AgNPs.

The elemental outline of the synthesized AgNPs (Figure 5) showed higher proportion of silver at 3 keV. Mostly, metallic silver nanoparticles display distinctive optical absorption peak almost at 3 keV due to their surface plasmon resonance.

### 3.5. Fourier Transform Infrared Spectroscopy

**Figure 6:**
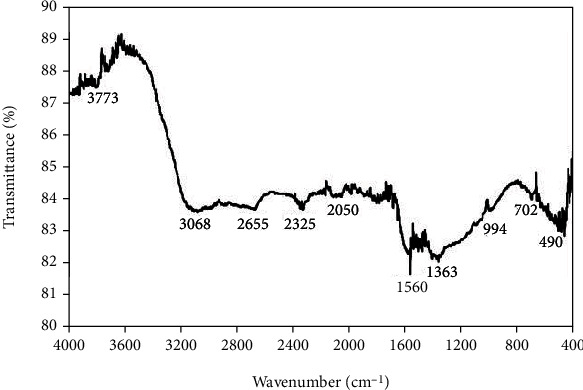
FT-IR spectrum of AgNPs synthesized using Fv-fucoidan, *X*-axis shows the wavenumber in cm^−1^, and *Y*-axis indicates the transmittance of the silver nanoparticles.

The inorganic biomolecules in the fucoidans responsible for the synthesis of silver nanoparticles were analyzed by using the FT-IR analysis (Figure 6). Silver nanoparticle-synthesized Fv-fucoidan had the absorption band at 3068 cm^−1^, showing C-H stretching groups of aromatics, and the low absorption band shows at 2655 and 2325 cm^−1^, indicating the presence of O-H stretching carboxylic groups. The band at 1560 cm^−1^ corresponds to amides of N-H bending group, 1363 cm^−1^ shows aliphatic nitro groups, and 994 cm^−1^ shows =C-H bending of alkenes groups. The small bands at 702 and 490 cm^−1^ indicate the presence of alkyl halides.

### 3.6. Antibacterial Activities of Fucoidan-Mediated Silver Nanoparticles

The result demonstrated that AgNPs display great bactericidal action against Gram-negative and Gram-positive microscopic organisms. The Gram-negative bacteria (*K. planticola*, *K. pneumoniae*, and *Serratia nematodiphila*) indicated bigger inhibition zones than the Gram-positive bacteria (*Bacillus Subtilis*, *Bacillus* sp., and *Streptococcus* sp.) (Table 1).

**Figure 7:**
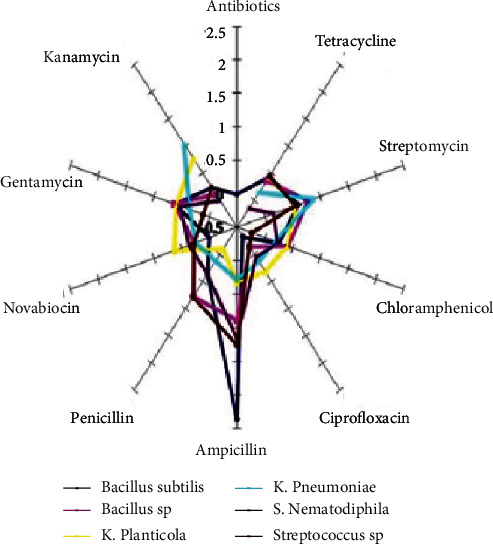
Increased fold area of fucoidan-stabilized AgNPs for enhanced antibacterial activity.

**Figure 8:**
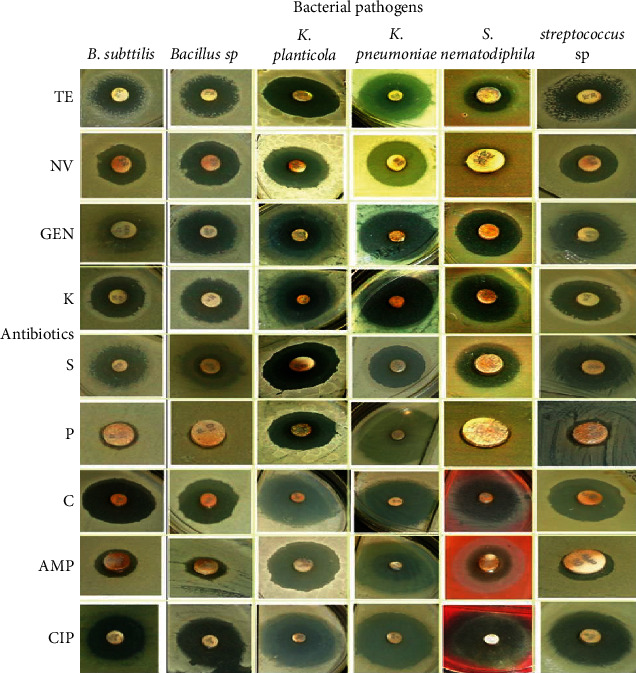
Enhanced antibacterial activity of the synthesized AgNPs impregnated with diverse antibiotics in the *Y*-axis the antibiotics such as TE (tetracycline), NV (novamycin), GEN (gentamycin), K (kanamycin), S (streptomycin), P (penicillin), C (cephalexin), AMP (ampicillin), CIP (ciprofloxacin) against the bacterial strains in *X*-axis *B. subtilis*, *Bacillus* sp., *K. planticola*, *K. pneumoniae*, *S. pnematodiphila*, and *Streptococcus* sp.

This might be due to the variety in the composition of their cell walls [14, 15]. The bactericidal action increases by increasing the AgNP concentration. Interestingly, the combination of AgNPs and diverse antibiotics demonstrated a synergistic impact. It was observed that AgNPs impregnated with novobiocin and penicillin disks showed a great inhibition zone (Table 2 and Figures 7 and 8) compared to the novobiocin and penicillin antibiotic-treated *S. nematodiphila* (6 mm), penicillin-treated *B. subtilis* (5 mm), *Bacillus* sp. (8 mm), and *Streptococcus* sp. (8 mm), and ampicillin-treated *B. subtilis* (11 mm) and *Streptococcus* sp. (9 mm).

### 3.7. Antifungal Activity of Fucoidan-Mediated Silver Nanoparticles

**Figure 9:**
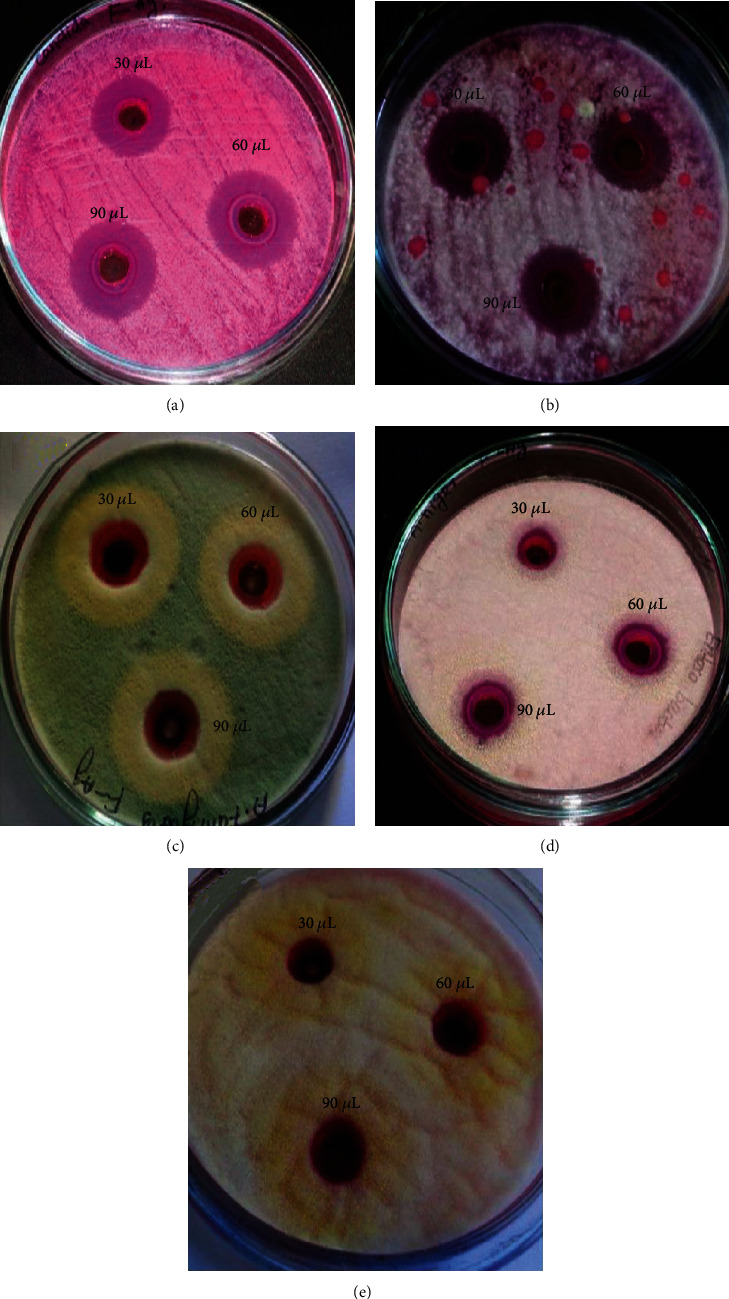
Fungicidal activity of the synthesized AgNPs against the selected fungi. (a) *C. albicans*, (b) *Fusarium* sp., (c) *A. fumigatus*, (d) *A. niger*, and (e) *A. flavus.*

**Figure 10:**
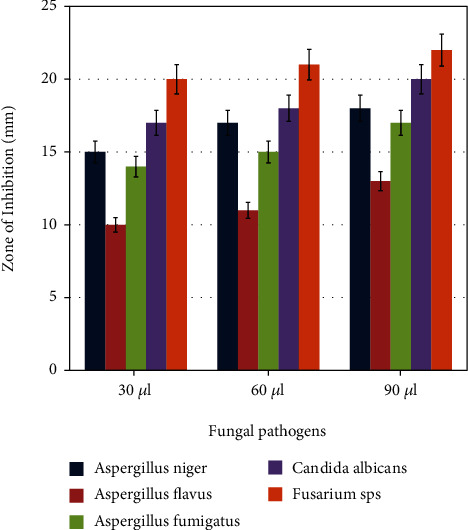
The fungicidal activity of AgNPs against different fungal pathogens. *X*-axis indicates the different concentration of silver nanoparticles, and *Y*-axis indicates the zone of inhibition in mm

The synthesized AgNPs showed excellent fungicidal activity against all chosen clinical isolates (Figures 9 and 10). The inhibition zone increases in a specific order ranging from *Fusarium* sp. (22.43 ± 0.296) > *C. albicans* (20.07 ± 0.067) > *A. niger* (17.87 ± 0.241) > *A. fumigatus* (17.50 ± 0.501) > *A. flavus* (12.97 ± 0.261). The minimal fungicidal action was noted against *A. flavus*.

### 3.8. Anticancer Activity of AgNPs against Liver and Lung Cancer Cell Lines

The liver and lung cancers are the most common cancers causing a lot of death globally. The results of anticancer action imply the high cytotoxic activity of the synthesized AgNPs against the tumor cells (HepG2 and A549) when compared with the standard cyclophosphamide. The cytotoxic effect of fucoidan-mediated silver nanoparticles was higher in A549 cell line than HepG2. As the concentration of AgNPs increases, the cytotoxicity also increases as predicted in Table 3.

### 3.9. DPPH Radical Scavenging Activity

**Figure 11:**
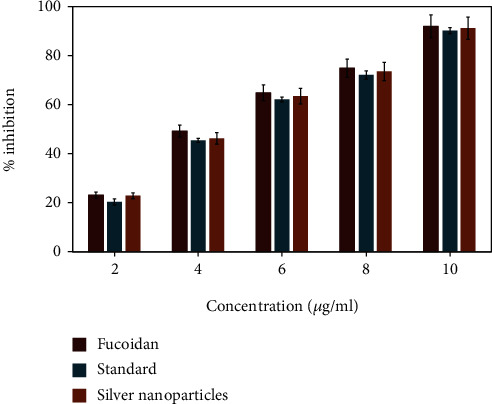
DPPH radical scavenging activity of fucoidan, fucoidan-stabilized AgNPs, and vitamin C as standard *X*-axis indicates the different concentrations of silver nanoparticles and *Y*-axis indicates the percentage of inhibition.

The free radical scavenging activity by the DPPH (2,2-diphenyl-2-picrylhydrazyl) method showed higher activity in the green synthesized AgNPs when compared with fucoidan and vitamin C (standard). The antioxidant activity of AgNPs displayed significant dosage-dependent inhibition (Figure 11). The maximum inhibition percentage of AgNPs at 10 *μ*g/ml concentration was recorded to be 84% for DPPH scavenging.

## 4. Discussion

Noble metals are known to exhibit unique optical properties due to the excitation of SPR. A distinctive surface plasmon resonance (SPR) band was observed at 430 nm after 24 h which is characteristic for AgNPs. As the incubation time was increased, the absorbance of SPR band increased too without band shift. This clearly reflects the development of AgNPs. The stability of the formed AgNPs was evidenced by measuring its UV-Vis absorbance after two months where no alteration in absorbance or shapes of bands was recognized. In the XRD results, the intensity of these peaks (111), (200), (220), and (311) reflects the high degree of crystalline nature of the AgNPs [11].

The EDX spectrum additionally shows that the weak signals for oxygen, nitrogen, and carbon were observed. This could be due to the biochemical molecules of Fv-fucoidan responsible for AgNP synthesis and stabilization [14]. In concurrence with previous studies, the AgNPs impregnated with antibiotics showed fine antibacterial action when compared with AgNPs and antibiotics separately [16, 17]. These are essential results to prompt a diminishment in the amount of medications important to treat illnesses, subsequently reducing side effects and improvement of antibacterial action against drug-resistant bacterial strains.

Despite the fact that it is more pathogenic, it also has more noteworthy harmfulness and produces mycotoxins. Moreover, the increase in AgNPs concentration reduces the antifungal action in a dose-dependent manner [35]. The cytotoxic effect of AgNPs is due to the physicochemical interaction of silver atoms with the functional groups of intracellular proteins, as well as with the phosphate group's nitrogen bases in DNA [2]. Some of the approved chemotherapeutic agents were identified to cause side effects. Accordingly, there is an imperative need to create alternative drugs against these deadly diseases. Thus, the green synthesized AgNPs from fucoidan extract act as a powerful free radical scavenger and thus establish their therapeutic importance [11, 35].

## 5. Conclusion

In the current study, we have recognized an ecofriendly, cost-effective, and facile method for the synthesizing AgNPs using fucoidan extract from *Fucus vesiculosus* source (brown marine algae) at ambient temperature as an effective green reducing and stabilizing agent. The spectroscopic characterization methods display the formation of stable, crystalline, and spherical shape of AgNPs with size ranges from 4 to 45 nm. The synthesized AgNPs show good antimicrobial activity against the selected six pathogenic microorganisms. They also showed a synergistic effect on the antimicrobial activity of the standard antibiotics. Moreover, AgNPs inhibit the cell viability of liver cancer cells lines (HepG2) and lung cancer cell lines (A549). In vitro antioxidant assays illustrated that AgNPs have the potential scavenging activity against DPPH. The conventional chemical methods for synthesizing nanoparticles are costly and time consuming and pose a great threat when disposed in environment, and contact with these chemically synthesized nanoparticles might result in major diseases like skin cancer and lung cancer. Therefore, this green synthesis approach appears to be an ecofriendly alternative to the conventional chemical methods and stands as an effective alternative drug that can be used for biomedical applications in the future.

## Figures and Tables

**Table 1:** Antibacterial activity of fucoidan-stabilized AgNPs.

AgNPs conc.	25 *μ*l	50 *μ*l	75 *μ*l
Bacterial isolates	Zone of inhibition (mm)		
*Bacillus subtilis*	09.03 ± 0.033	10.17 ± 0.088	11.17 ± 0.088
*Bacillus* sp.	09.27 ± 0.146	09.97 ± 0.033	11.03 ± 0.033
*K. planticola*	09.07 ± 0.120	10.07 ± 0.120	11.77 ± 0.394
*K. pneumoniae*	10.23 ± 0.186	11.00 ± 0.000	15.53 ± 0.318
*Serratia nematodiphila*	10.20 ± 0.116	11.00 ± 0.000	14.00 ± 0.000
*Streptococcus* sp.	09.06 ± 0.177	09.67 ± 0.334	10.23 ± 0.234

**Table 2:** Antibacterial activity of diverse antibiotics and AgNPs impregnated with diverse antibiotics (blend).

Bacterial isolates	*Bacillus subtilis*		*Bacillus* sp.		*K. planticola*		*K. pneumoniae*		*Serratia nematodiphila*		*Streptococcus* sp.	
	Zone of inhibition (mm)											
Antibiotics	Ab	Blend	Ab	Blend	Ab	Blend	Ab	Blend	Ab	Blend	Ab	Blend
Tetracycline	15	18	18	21	21	25	29	31	13	12	15	18
Novobiocin	15	15	16	18	18	23	18	20	0	6	15	17
Gentamicin	11	14	18	23	19	24	25	29	17	21	19	20
Kanamycin	18	20	19	20	21	28	21	30	19	19	18	20
Streptomycin	15	19	15	20	16	20	16	22	13	14	16	20
Penicillin	0	5	0	8	20	19	36	38	0	6	0	8
Chloramphenicol	25	28	21	25	32	38	34	37	27	30	24	21
Ampicillin	0	11	8	11	20	23	38	43	15	22	0	9
Ciprofloxacin	27	22	27	25	34	39	31	33	40	41	27	25

**Table 3:** Anticancer activity of AgNPs against liver (HepG2) and lung (A549) cancer cell lines.

	AgNPs conc. (*μ*g)	1	10	25	50	100
HepG2	% cell viability (treatment)	90.48	74.23	59.25	47.56	32.22
	% cell viability (cyclophosphamide)	87.81	73.40	23.21	1.93	1.48

A549	% cell viability (treatment)	98.27	89.26	75.20	68.25	50.14
	% cell viability (cyclophosphamide)	85.57	68.64	37.90	19.30	5.40

## Data Availability

The authors confirm that the data supporting the findings of this study are available within the article.
